# Supplementary material for: Abalone adhesion: The role of various adhesion forces and their proportion to total adhesion force
Source: PLoS One. 2023 Jun 9;18(6):e0286567. doi: 10.1371/journal.pone.0286567 (PMC10256213; doi:10.1371/journal.pone.0286567)
Supplement: Attachment — Submitted filename: Response to Reviewers.docx [file pone.0286567.s001.docx]

**Response to Reviewers**

Additional Editor Comments:

1. Please follow the Journal style of Plos One for writing-up, references and citations in the context.

I have revised writing-up, references and citations in the manuscript according to the Journal style of Plos One.

2. Some subtitles are too general. Please use more exact subtitles to reflect the context in that section. All subtitles should be consistent in the whole manuscript

I have revised some subtitles.

3. Please clearly define your novelty or contributions of this manuscript at the right positions. Please indicate the sources of formulae if necessary and state the calculation procedure.

I have defined my novelty or contributions of this manuscript at the right positions.

I have added the sources of formulae and state the calculation procedure at the right positions of manuscript.

4. Current reference list seems too old. The newest ones are in 2018. Please update the reference list by adding more references within the last three years. These references can help you to define the problem.

I have added more references within the last three years and update the reference list.
