# Supplementary material for: Abalone adhesion: The role of various adhesion forces and their proportion to total adhesion force
Source: PLoS One. 2023 Jun 9;18(6):e0286567. doi: 10.1371/journal.pone.0286567 (PMC10256213; doi:10.1371/journal.pone.0286567)
Supplement: Attachment — Submitted filename: Response to Reviewers.docx [file pone.0286567.s002.docx]

**Response to Reviewers**

Review Comments to the Author

Minor points:

1. The mucus of the abalone gastropod can affect the observation of the gastropod microstructure. How to remove it?

In the pretreatment process of microscopic observation of abalone gastropod samples by SEM, the abalone gastropod sample was rinsed with phosphate buffer (PBS) for 3 times, each time for about 15min. This process can remove the mucus on the surface of the gastropod.

2. Abalone is alive and the adhesion force of the abalone will suddenly increase under external stimulation. How to consider the effect of biological activity on adhesion results?

During the tensile test of abalone, the force measuring plate and abalone adhere on its surface are put on the test machine. The force measuring plate is fixed first, and then the abalone shell is hooked with the hook. The lifting speed of the tensile testing machine is 100mm/min.

When the abalone shell is hooked with the hook, the abalone is stimulated. Due to the instinct of the creature to seek advantages and avoid harm, the adhesion force between the gastropod and the force measuring plate suddenly increases. At this time, the test machine has not started. When the test machine is started, the speed of the test machine stretching upward is very slow (100mm/min), so that the abalone has enough reaction time to further improve the adhesion force between the gastropod and the force measuring plate to resist the upward tension. Therefore, the adhesion force results obtained in the tensile test have taken into account the effect of biological activity on the adhesion results.

At the same time, the biological activity of each abalone has a slight difference effects on the adhesion force results. Therefore, this study reduced the effect of the difference in adhesion force results by averaging multiple tests.

3. The authors mentioned that “the dead abalone abdominal foot does not produce vacuum adhesion force and van der Waals force” in line 282. Why does the van der Waals force disappear with the death of abalone?

The van der Waals force is generated when a large amount of fibers on the surface of the abalone gastropod come into contact with the force measuring plate. When the abalone is alive, the fibers on the surface of the gastropod are also active and flexible. Therefore, when abalone is placed on the force measuring plate, the fibers distribution at the microscopic level can change with the change of the surface morphology of the force measuring plate, and the top of the fibers can effectively contact with the force measuring plate to generate van der Waals force.

When the abalone died, the surface of its gastropod contacted with the force measuring plate did not produce van der Waals force.

The dead abalone test sample used in this experiment was obtained from the force measuring plate after the abalone died naturally on the force measuring plate. When the abalone died, the fibers on the surface of the gastropod also lost their life activity, and the fibers became hard and lost their flexibility. The fibers on the gastropod are largely lodging, making it difficult to form an upright state. Therefore, when abalone is placed on the force measuring plate, the fibers distribution at the microscopic level can not change with the change of the surface morphology of the force measuring plate, and the top of the fibers can not effectively contact with the force measuring plate to generate van der Waals force.

4. The adhesion force survey of abalone and leech is not sufficient. See:

[1] Li et al., Hard to be killed: Load-bearing capacity of the leech Hirudo nipponia. Journal of the Mechanical Behavior of Biomedical Materials, 2018.

[2] Zhang et al., A Mechanics Study on the Self-Righting of Abalone from the Substrate. Applied Bionics and Biomechanics, 2020

I have added the corresponding references in the text.

5. The sentence in L14 to L15 needs revising.

I have made corresponding modifications to the sentence in L14 to L15.

6. Between the number and its unit, there should be a blank.

I have checked the full text and added a blank between the number and its unit.

7. The symbol “;” is seldom used in English articles.

I have checked the full text and modified the symbol “;”

8. The mechanical parameters should be correctly written. If they are varibles, they should be in the italic format.

I have checked the full text and modified the variables to italic format.

9. The symbol “Pi” in Eq. (1) is a constant, and it is not italic.

I have made corresponding modifications.
