# Supplementary material for: Abalone adhesion: The role of various adhesion forces and their proportion to total adhesion force
Source: PLoS One. 2023 Jun 9;18(6):e0286567. doi: 10.1371/journal.pone.0286567 (PMC10256213; doi:10.1371/journal.pone.0286567)
Supplement: Attachment — Submitted filename: Response to Reviewers.docx [file pone.0286567.s003.docx]

**Response to Reviewers**

Minor points:

The authors corrected all the necessary corrections. However, in the abstract, the results are not included. There should be some quantitative outcomes.

I have added some quantitative outcomes in the abstract.
